# Cerebral Perfusion Patterns of Anxiety State in Patients With Pulmonary Nodules: A Study of Cerebral Blood Flow Based on Arterial Spin Labeling

**DOI:** 10.3389/fnins.2022.912665

**Published:** 2022-05-09

**Authors:** Xiao-Hui Wang, Xiao-Fan Liu, Min Ao, Ting Wang, Jinglan He, Yue-Wen Gu, Jing-Wen Fan, Li Yang, Renqiang Yu, Shuliang Guo

**Affiliations:** ^1^Department of Pulmonary and Critical Care Medicine, The First Affiliated Hospital of Chongqing Medical University, Chongqing, China; ^2^School of Medical Technology, Shaanxi University of Chinese Medicine, Xianyang, China; ^3^Department of Psychiatry, The First Affiliated Hospital of Chongqing Medical University, Chongqing, China; ^4^Department of Clinical Psychology, Fourth Military Medical University, Xi’an, China; ^5^Department of Radiology, The First Affiliated Hospital of Chongqing Medical University, Chongqing, China

**Keywords:** pulmonary nodules, anxiety, arterial spin labeling, cerebral blood flow, cerebral perfusion

## Abstract

**Background and Purpose:**

The proportion of patients with somatic diseases associated with anxiety is increasing each year, and pulmonary nodules have become a non-negligible cause of anxiety, the mechanism of which is unclear. The study focus on the cerebral blood flow (CBF) of anxiety in patients with pulmonary nodules to explore the cerebral perfusion pattern of anxiety associated with pulmonary nodules, blood perfusion status and mode of pulmonary nodule induced anxiety state.

**Materials and Methods:**

Patients with unconfirmed pulmonary nodules were evaluated by Hamilton Anxiety Scale (HAMA). The total score > 14 was defined as anxiety group, and the total score ≤ 14 points was defined as non-anxiety group. A total of 38 patients were enrolled, of which 19 patients were the anxiety group and 19 were the non-anxiety group. All subjects underwent arterial spin labeling imaging using a 3.0 T MRI. A two-sample *t*-test was performed to compare the CBF between the two groups. The CBF was extracted in brain regions with difference, and Spearman correlation was used to analyze the correlation between CBF and HAMA scores; ROC was used to analyze the performance of CBF to distinguish between the anxiety group and the non-anxiety group.

**Results:**

The CBF in the right insula/Heschl’s cortex of the anxiety group decreased (cluster = 109, peak *t* = 4.124, and *P* < 0.001), and the CBF in the right postcentral gyrus increased (cluster = 53, peak *t* = −3.912, and *P* < 0.001) in the anxiety group. But there was no correlation between CBF and HAMA score. The ROC analysis of the CBF of the right insula/Heschl’s cortex showed that the AUC was 0.856 (95%CI, 0.729, 0.983; *P* < 0.001), the optimal cutoff value of the CBF was 50.899, with the sensitivity of 0.895, and specificity of 0.789. The ROC analysis of CBF in the right postcentral gyrus showed that the AUC was 0.845 (95%CI, 0.718, 0.972; *P* < 0.001), the optimal cutoff value of CBF was 43.595, with the sensitivity of 0.737, and specificity of 0.842.

**Conclusion:**

The CBF of the right insula/Heschl’s cortex decreased and the CBF of the right postcentral gyrus increased in patients with pulmonary nodules under anxiety state, and the CBF of the aforementioned brain regions can accurately distinguish the anxiety group from the non-anxiety group.

## Introduction

Anxiety disorder is a group of mental disorders that mainly manifested by pathological anxiety symptoms. According to the clinical manifestations and pathogenesis, it mainly includes generalized anxiety disorder (GAD), specific phobia (animal, natural environment and blood-injection-injury, etc.), panic disorder, anxiety disorders due to another medical condition, etc. In recent years, the proportion of patients with somatic diseases accompanied by anxiety disorders has increased significantly. The somatic diseases highly related to anxiety mainly include neurological diseases, non-cardiac chest pain, diabetes, gastrointestinal diseases, and cardiovascular and respiratory diseases. Somatic and mental disorders are inextricably linked (Wang et al., 2019). Psychological abnormalities caused by somatic diseases can lead to damage to social functions that required timely intervention. Currently, pulmonary nodules have become a non-negligible somatic disease of anxiety.

Low-dose computed tomography (LDCT) has been widely recommended to screen lung cancer for high risk adults (Zhou et al., 2018), and lung cancer mortality and overall mortality might decrease by 20 and 6.7%, respectively (Aberle et al., 2011). As the increasing number of people undergo LDCT screening, millions of people are found to have pulmonary nodules every year (Slatore and Wiener, 2018). However, the vast majority of pulmonary nodules are benign (Fan et al., 2019; Lin et al., 2019), individuals with which may experience psychological harm as a result of a “near-cancer” diagnosis (Slatore and Wiener, 2018; Li L. et al., 2020). Previous studies show the incidence of anxiety in patients with pulmonary nodules ranges from 39.8 to 59.3% (Freiman et al., 2016; Li L. et al., 2020; Wang et al., 2020). But the neural basis of anxiety in patients with pulmonary nodules is still complex and vague.

The neuroimaging, especially magnetic resonance imaging (MRI), may provide critical information for understanding the disease. The cerebral perfusion is fundamental for the function of brain, and perfusion imaging can be used to indirectly or directly understand these changes. Arterial spinal labeling (ASL) is a imaging method with greater reliability over blood oxygen level-dependent functional magnetic resonance imaging (BOLD-fMRI) that can measure the regional cerebral blood flow (CBF) quantitatively (Tuite, 2017). In recent years, CBF is closely related to normal brain function, so it has attracted much attention in the research of brain function, and may provide a large amount of brain physiological information (Liang et al., 2013), particularly in neurological and psychiatric disorders (Cui et al., 2017a,b; Yu et al., 2021; Chen et al., 2022; Hu et al., 2022; Wang et al., 2022). However, no study has researched the changes of CBF in anxiety of patients with pulmonary nodules.

Therefore, we conducted CBF research based on ASL to explore the cerebral perfusion pattern of anxiety associated with pulmonary nodules, blood perfusion status and mode of pulmonary nodule anxiety induced state, so as to contribute to a more comprehension of its neuroimaging phenotype and provide the basis and possibility for exploring new and more effective intervention measures.

## Materials and Methods

### Patients

From March 2021 to September 2021, 42 patients with unconfirmed pulmonary nodules from the Pulmonary and Critical Care Medicine, the First Affiliated Hospital of Chongqing Medical University underwent MRI. Four patients were excluded, two of which were found obvious demyelinating lesions, and the other 2 patients’ image quality could not meet the analysis standards. A total of 38 patients were included in this study, of which 19 patients entered the anxiety group and 19 entered the non-anxiety group. The patients of the two groups were matched for sex, age, and years of education. The inclusion criteria were as follows: (1) all patients were aged from 18 to 65 years old; (2) pulmonary nodules were found by chest CT examination without definite pathological diagnosis by puncture or surgery, diameter of which ≤3 cm; and (3) they were right handed. The exclusion criteria were as follows: (1) a history of mental illness; (2) communication impairments, such as hearing or speech impairments; (3) suffering from a severely unstable physical condition; (4) combined with hypertension, diabetes, thyroid disease, heart disease, chronic obstructive pulmonary disease, bronchiectasis, narrow-angle glaucoma, previous head trauma, cerebral infarction, cerebral hemorrhage, intracranial mass, epilepsy, disturbance of consciousness, and other intracranial organic lesions; (5) pregnant or breast-feeding women, or those planning to become pregnant; (6) presence of fixed dentures or tattoos; (7) MRI examination revealed intracranial dysplasia, mass, severe multiple lacunar infarction and demyelinating lesions; (8) metal implants in the body, such as the skull and mouth, which affect the quality of image data; and (9) contraindications to MRI scanning.

The demographic characteristics of the patients were collected, including gender, age, ethnicity, marital status, years of education, occupation, alcohol consumption, smoking history, second-hand smoke exposure, malignant tumor history, and family history of malignancy. The clinical data were collected, including respiratory symptoms, course of pulmonary nodules, number of pulmonary nodules, diameter of the largest pulmonary nodule, and the decision of doctors. The written informed consent of all individuals were obtained. The study was approved by the institutional of the First Affiliated Hospital of Chongqing Medical University Ethics Board (2019-083).

### Clinical Assessment

Patients were evaluated by Hamilton Anxiety Scale (HAMA) in a quiet studio before undertaking MRI. The HAMA is a 14-item rating scale that measures the severity of anxiety based on the frequency and impairment of symptoms during the past week. Each item ranges from 0 (not present) to 4 (very severe). Higher scores indicate a greater degree of anxiety. The total score > 14 was defined as anxiety group, and the total score ≤ 14 points was defined as non-anxiety group.

### Image Acquisition

The subjects were required to lie down quietly and with their eyes closed. During the scanning process, they were required to stay awake and not fall asleep. Both ears were plugged with earplugs to protect their hearing. The head was fixed with a sponge to reduce head movement. A standard 8-channel head coil was used with a GE signal Signa HDx 3.0 T MRI scanner (United States). Intracranial lesions and structural abnormalities were excluded using 3D high-resolution T1WI, T2WI, and T2 FLAIR sequences, followed by ASL. ASL parameters: TR 4639 ms, TE 9.8 ms, slice thickness 4.0 mm, acquisition slices 40, NEX 3, FOV 24 mm × 240 mm, matrix 512 × 8 (3D spiral filling), PLD 1525 ms, acquisition time 4 min 20 s.

### Image Processing

The DICOM images were converted into NIFTI format by dcm2nii software, and then SPM12 was run in the Matlab13b, and the image of each subject was registered by the one-step registration method, and the image quality after registration was checked. Then, dpabi4.3 was used for image normalization; SPM12 was used for spatial smoothing, and the full width at half-maximum of the Gaussian kernel was 6 mm. As performed by a previous study (He et al., 2019), the data were standardized by the signal of whole brain.

### Statistical Analysis

Two-sample *t*-test was used to compare CBF between groups, and age, gender, and years of education were used as covariates for regression. The *P* value was set as 0.001 (cluster > 50). The above steps was completed based on SPM12. Xjview10 was used to visualize the results, combined with DPABI 4.3 to extract the CBF values of the brain regions with difference. Spearman correlation analysis was used to evaluate the correlation between the CBF value and HAMA score; ROC analysis was used to assess the capacity of the CBF for distinguishing the anxiety group from the non-anxiety group, and *P* < 0.05 were considered statistically significant.

## Results

### Demographic and Clinical Data

The proportion of single pulmonary nodules in the anxiety group was significantly lower than that in the non-anxiety group (15.79% vs. 47.37%, *P* = 0.039). There were no significant difference between the two groups in gender, marital status, daily alcohol consumption, smoking history, secondhand smoke exposure history, malignant tumor history, tumor family history, occupational exposure history, pulmonary nodules detected by physical examination, respiratory symptoms, decision of doctors, age, years of education, course of pulmonary nodules, and the diameter of the largest pulmonary nodule (*P* > 0.05, Table 1).

**TABLE 1:** Comparison of demographic characteristics and clinical data between anxiety group and non-anxiety group.

	Anxiety group	Non-anxiety group	χ^2^ or *t* or Z value	*P* value
**Gender**				
Female, *n* (%)	13 (68.42%)	11 (57.89%)	0.440	0.507
Male, *n* (%)	6 (31.58%)	8 (42.11%)		
**Marital status**				
Married, *n* (%)	15 (78.95%)	18 (94.74%)	2.018	0.155
No spouse, *n* (%)	4 (21.05%)	1 (5.26%)		
**Daily alcohol consumption**				
Yes, *n* (%)	1 (5.26%)	1 (5.26%)	0.000	1.000
No, *n* (%)	18 (94.74%)	18 (94.74%)		
**Smoking**				
Yes, *n* (%)	4 (21.05%)	4 (21.05%)	0.000	1.000
No, *n* (%)	15 (78.95%)	15 (78.95%)		
**Malignant tumor history**				
Yes, *n* (%)	1 (5.26%)	0 (0.00%)	1.000	0.317
No, *n* (%)	18 (94.74%)	19 (100.00%)		
**Family history of tumor**				
Yes, *n* (%)	9 (47.37%)	8 (42.11%)	0.104	0.748
No, *n* (%)	10 (52.63%)	11 (57.89%)		
**Occupational exposure history**				
Yes, *n* (%)	2 (10.53%)	0 (0.00%)	2.056	0.152
No, *n* (%)	17 (89.47%)	19 (100.00%)		
**Secondhand smoke exposure history**				
Yes, *n* (%)	8 (42.11%)	3 (15.79%)	3.114	0.078
No, *n* (%)	11 (57.89%)	16 (84.21%)		
**Pulmonary nodules detected by physical examination**				
Yes, *n* (%)	13 (68.42%)	13 (68.42%)	0.000	1.000
No, *n* (%)	6 (31.58%)	6 (31.58%)		
**Respiratory symptoms**				
Yes, *n* (%)	3 (15.79%)	3 (15.79%)	0.000	1.000
No, *n* (%)	16 (84.21%)	16 (84.21%)		
**Numbers of pulmonary nodules**				
Single, *n* (%)	3 (15.79%)	9 (47.37%)	4.269	0.039
≥2 nodules, *n* (%)	16 (84.21%)	10 (52.63%)		
**Decision of doctors**				
Follow-up, *n* (%)	12 (63.16%)	16 (84.21%)	2.114	0.146
Puncture or surgery, *n* (%)	7 (36.84%)	3 (15.79%)		
Age (years),−x ± *s*	48.58 ± 7.11	43.37 ± 10.67	1.771	0.086
Years of education, M (Q1, Q3)	12 (9, 15)	14 (10, 15)	–0.747	0.455^&^
Course of pulmonary nodules (month), M (Q1, Q3)	4 (1, 12)	8 (1, 17)	–0.927	0.354^&^
The diameter of the largest pulmonary nodule (mm), M (Q1, Q3)	5 (4, 10)	5 (4, 7)	–0.695	0.487^&^

*“^&^” Years of education, course of pulmonary nodules, and the diameter of the largest pulmonary nodule showed non-normal distribution, using Mann–Whitney U test.*

### Difference of Cerebral Blood Flow Between Anxiety Group and Non-anxiety Group

**FIGURE 1:**
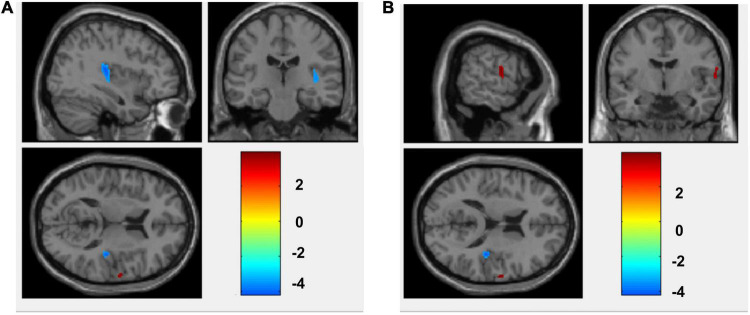
CBF differences between anxiety and non-anxiety group patients with pulmonary nodules. **(A)** The blue area represents CBF decrease (Insula_R/Heschl_R). **(B)** The red area represents CBF increase (Postcentral_R).

**FIGURE 2:**
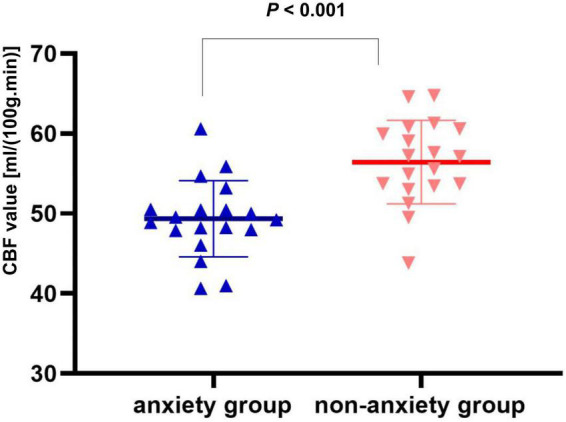
Two-sample *t* test of CBF in the right insula/Heschl’s cortex between anxiety group and non-anxiety group.

**FIGURE 3:**
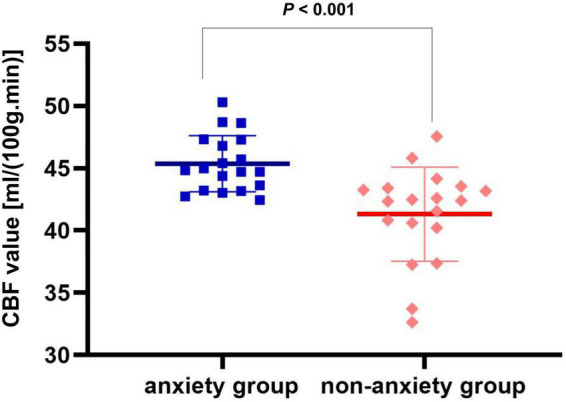
Two-sample *t* test of CBF in the right postcentral gyrus between anxiety group and non-anxiety group.

Compared with the non-anxiety group, CBF decreased in the right insula/Heschl’s cortex (cluster = 109, peak *t* = 4.124, and *P* < 0.001; Figure 1A), and increased in the right postcentral gyrus (cluster = 53, peak *t* = −3.912, and *P* < 0.001; Figure 1B) in anxiety group. Two-sample *t*-test revealed that CBF in the right insula/Heschl’s cortex of anxiety group was significantly lower than non-anxiety (49.34 ± 4.76 vs. 56.42 ± 5.21, *t* = −4.374, and *P* < 0.001; Figure 2), while CBF in the right postcentral gyrus of anxiety group was significantly higher than non-anxiety (45.37 ± 2.25 vs. 41.31 ± 3.77, *t* = 4.024, and *P* < 0.001; Figure 3).

### Correlation Between Cerebral Blood Flow and Hamilton Anxiety Scale Score

**FIGURE 4:**
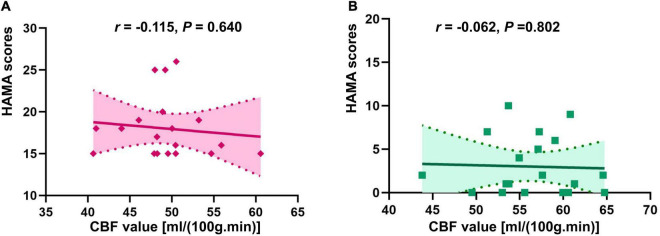
Correlation analysis of CBF in brain regions with difference (Insula_R/Heschl_R) and HAMA score in two groups. **(A)** The correlation analysis in anxiety group. **(B)** The correlation analysis in non-anxiety group.

**FIGURE 5:**
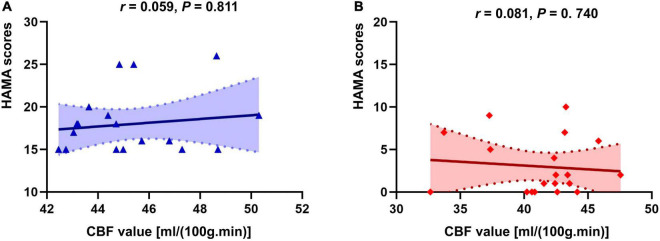
Correlation analysis of CBF in brain regions with difference (Postcentral_R) and HAMA score in two groups. **(A)** The correlation analysis in anxiety group. **(B)** The correlation analysis in non-anxiety group.

There was no correlation of CBF in the right insula/Heschl’s cortex and HAMA in both anxiety and non-anxiety groups (*r* = −0.115, *P* = 0.640; *r* = −0.062, *P* = 0.802; Figure 4). There was no correlation between CBF in the right postcentral gyrus with difference and HAMA in both anxiety and non-anxiety groups (*r* = 0.059, *P* = 0.811; *r* = 0.081, *P* = 0.740; Figure 5).

### Capacity of Cerebral Blood Flow in Anxiety Classification

**FIGURE 6:**
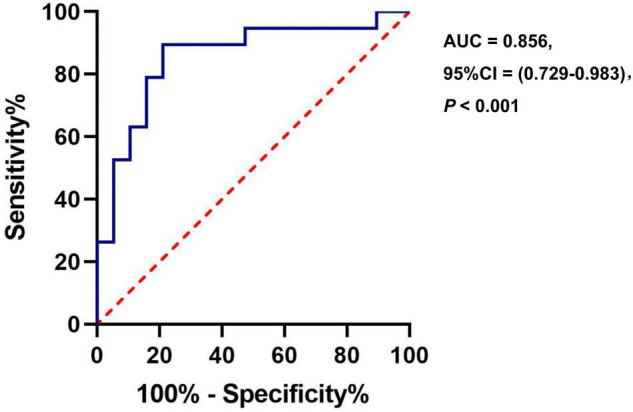
The ROC curve of CBF in the brain regions with difference (Insula_R/Heschl_R) for distinguishing the anxiety group from the non-anxiety group.

**FIGURE 7:**
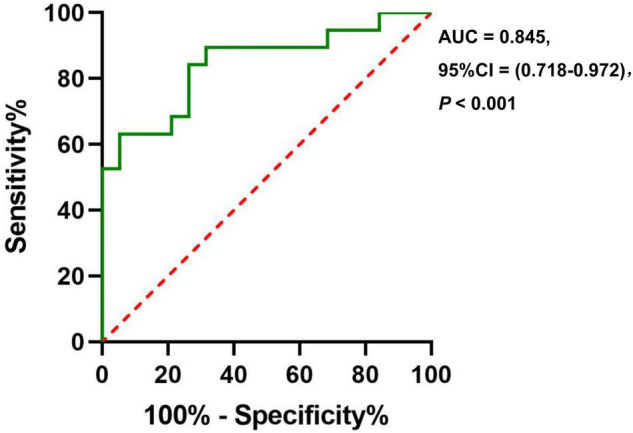
The ROC curve of CBF in the brain regions with difference (Postcentral_R) for distinguishing the anxiety group from the non-anxiety group.

The ROC analysis of CBF in the right insula/Heschl’s cortex showed an AUC of 0.856 (95%CI, 0.729, 0.983; *P* < 0.001). When the optimal cutoff value of CBF was 50.899, the Youden index was 0.684, the sensitivity was 0.895, the specificity was 0.789, and the accuracy was 0.821 (Figure 6). The ROC analysis of CBF in the right postcentral gyrus showed an AUC of 0.845 (95%CI, 0.718, 0.972; *P* < 0.001). When the optimal cutoff value of CBF was 43.595, the Youden index was 0.579, the sensitivity was 0.737, the specificity was 0.842, and the accuracy was 0.789 (Figure 7).

## Discussion

In this study, we performed ASL imaging and calculated CBF. It was found that in patients with pulmonary nodules combined with anxiety, the CBF of the right insula/Heschl’s cortex decreased, and the CBF of the right postcentral gyrus increased. And the CBF values of the above-mentioned brain regions with difference can accurately distinguish the anxiety group from the non-anxiety group. ASL is a non-invasive imaging technique that can quantitatively reflect tissue perfusion. Blood flow is the blood delivered to a unit of tissue per unit time, reflecting the metabolic and functional state of the tissue. It has been used for the research in brain tumors (Batalov et al., 2021), vascular cognitive impairment (Malojcic et al., 2017), brain injury (Li N. et al., 2020), ischemic stroke (Aracki-Trenkic et al., 2020), depression (Zhang et al., 2020), Alzheimer’s disease (Malojcic et al., 2017), kidney disease (Nery et al., 2019), musculoskeletal tumor (Xu et al., 2018), and even lung cystic fibrosis (Hernandez-Garcia et al., 2019), at the same time, it has also been widely used in clinical practice.

From a cognitive neuroscience perspective, anxiety is a state of distress and arousal prototypically evoked by uncertain danger (Hur et al., 2020), and anxiety disorder is the most common familial mental disorder (Collaborators, 2016). Persistent state of anxiety can cause social and physical dysfunction sometimes (Conway et al., 2019). Existing treatments still need to improve on the effectiveness and the side effects (Griebel and Holmes, 2013). Therefore, it is urgent to explore its neurobiological mechanism further. Previous studies have proved the high incidence of anxiety in patients with pulmonary nodules (Freiman et al., 2016; Li L. et al., 2020; Wang et al., 2020), so pulmonary nodules have become a non-negligible cause of anxiety, the CBF changes of which is still unclear.

This study has explored the CBF in anxiety patients with pulmonary nodules and found decreased CBF in the right insula/Heschl’s cortex and increased CBF in the right postcentral gyrus. Although there was no correlation of CBF in the aforementioned brain regions and HAMA scores, they contribute to the differentiation of anxiety group from the non-anxiety group. When the CBF value was selected at the optimal cutoff point, the accuracy of CBF in right insula/Heschl’s cortex is higher than right postcentral gyrus.

Cerebral blood flow has been used to investigate the neurobiological mechanisms of other anxiety disorders. Anxiety may lead to poor performance in cognitive tests and neuroimaging differences in patients with somatic diseases. Studies have shown that individuals with comorbid symptoms of anxiety and depression have lower performance in all cognitive tests, and reduced CBF in gray matter (Raffield et al., 2016). Anxiety and depression are the most common symptoms in neuropsychiatric systemic lupus erythematosus (NPSLE; Moustafa et al., 2020), that may have significant impact on mental health and health-related quality of life. Reduced prefrontal white matter perfusion was reported in a group of patients with NPSLE, which was associated with more severe anxiety symptoms (Papadaki et al., 2019). The frontal lobe is also considered to be an essential part of emotion assessment and regulation networks (Dixon et al., 2017).

Further studies have shown that individual differences in anxiety symptoms in NPSLE are related to specific and complex hemodynamics in the limbic system and prefrontal brain regions, as anxiety symptoms are mainly related to the increased perfusion dynamics in the right amygdala (Antypa et al., 2021). This study found changes in the CBF of the right insula/Heschl’s cortex and postcentral gyrus in anxiety patients with pulmonary nodules, of which the insula is currently considered to belong to the limbic system. The connectivity of subcortical areas to insula plays a pivotal role in anxiety (Robinson et al., 2019). However, the present study did not find abnormal perfusion in the frontal lobe of patients with pulmonary nodules combined with anxiety, which may be related to different underlying diseases.

A previous study reported increased CBF in the posterior temporal-occipital area of patients with GAD when dealing with sentences induced anxiety, meanwhile, no significant between-group difference was observed between the patients and healthy control (Andreescu et al., 2011). In addition, when patients with GAD were instructed to suppress their worries through reassessment, reduced CBF was found in the striatum, prefrontal cortex, middle cingulate, and sensorimotor areas (Karim et al., 2017a). In patients with chronic traumatic brain injury, voxel-wise analysis showed that CBF in the hippocampus, parahippocampus, rostral anterior cingulate, inferior frontal gyrus, and other temporal regions were negatively associated with self-reported anxiety. Moreover, region of interest (ROI) analysis revealed that CBF in hippocampal and rostral anterior cingulate were negatively associated with symptoms of anxiety (Thomas et al., 2021).

Notably, the changed brain areas are not common areas in anxiety disorders-related studies. The subcortical areas (the bed nucleus of the stria terminalis, the amygdala, and the hippocampus) are highlighted in the neural circuitry of anxiety (Robinson et al., 2019). However, somatic diseases are risk factors for developing anxiety symptom by causing neurohumoral dysfunction, and there is a bidirectional association between anxiety and somatic disease (Henning et al., 2020). The underlying mechanisms of comorbid anxiety in individuals with somatic diseases comprise unhealthy lifestyles, low treatment adherence, and dysregulations of psychobiological stress systems (Penninx et al., 2021), differing from anxiety disorders. As the first research of CBF in patients with pulmonary nodules complicated with anxiety, it was found that CBF decreased in the right insula/Heschl’s cortex and increased in the right postcentral gyrus, which is inconsistent with the abnormal CBF brain region found in the above study, suggesting that patients with pulmonary nodules complicated with anxiety have a different cerebral perfusion pattern from other anxious people. But, in another study revealed group differences in CBF in the inferior parietal lobule and in the postcentral and precentral gyri during anxiety induction of patients (Karim et al., 2017b), and the CBF alteration in the postcentral gyrus was consistent with the present study, suggesting a common neuroimaging phenotype in certain types of anxiety.

However, the mechanisms of anxiety are quite complex. Lots of studies show that anxiety-fear stimuli affect multiple regions, including the anterior insula, middle cingulate cortex, thalamus, and amygdala (Fullana et al., 2018). A meta-analysis showed that both induced anxiety and pathological anxiety showed increased activity in the left and right insula and cingulate cortex/medial prefrontal cortex; however, When the analyses were split by disorder, specific phobia appeared the most, and GAD the least, similar to induced anxiety (Chavanne and Robinson, 2021). Even Murty et al. (2022) found that the effect of anxious apprehension was distributed across the brain and that the temporal evolution of the responses was quite varied, including more transient and more sustained profiles, as well as signal increases and decreases with threat. Hence, more researches need to be performed to validate the results.

There are some limitations of our study. First, a single-modality study sequence was used in our study. Diffusion tensor imaging and fMRI will be needed in the future. Second, the sample size was not large. Although our study was able to detect clinically relevant effects, it is important to conduct large sample research in the future.

## Conclusion

Therefore, decreased CBF in the right insula cortex of the anxiety group found by the current study demonstrates the neurobiological basis of the anxiety state to some extent. CBF in the right insula/Heschl’s cortex and right postcentral gyrus has a capability to distinguish anxiety group from the non-anxiety group. This study broadens the perspective for analyzing the brain phenotype of patients with pulmonary nodules complicated with anxiety.

## Data Availability Statement

The raw data supporting the conclusions of this article will be made available by the authors, without undue reservation.

## Ethics Statement

The studies involving human participants were reviewed and approved by the institutional of the First Affiliated Hospital of Chongqing Medical University Ethics Board. The patients/participants provided their written informed consent to participate in this study.

## Author Contributions

X-HW and SG contributed to the conception and design of the study. X-HW and X-FL were in charge of the manuscript draft. X-HW, RY, TW, and MA collected and confirmed data and image accuracy. LY was responsible for the management of pulmonary nodules. JH was responsible for anxiety evaluation and consultation. X-HW, Y-WG, J-WF, and RY were responsible for statistical analysis of data. All authors made substantial revisions to the manuscript and approved the submitted revision.

## Conflict of Interest

The authors declare that the research was conducted in the absence of any commercial or financial relationships that could be construed as a potential conflict of interest.

## Publisher’s Note

All claims expressed in this article are solely those of the authors and do not necessarily represent those of their affiliated organizations, or those of the publisher, the editors and the reviewers. Any product that may be evaluated in this article, or claim that may be made by its manufacturer, is not guaranteed or endorsed by the publisher.
